# Evaluation of Scalability and Degree of Fine-Tuning of Deep Convolutional Neural Networks for COVID-19 Screening on Chest X-ray Images Using Explainable Deep-Learning Algorithm

**DOI:** 10.3390/jpm10040213

**Published:** 2020-11-07

**Authors:** Ki-Sun Lee, Jae Young Kim, Eun-tae Jeon, Won Suk Choi, Nan Hee Kim, Ki Yeol Lee

**Affiliations:** 1 Medical Science Research Center, Ansan Hospital, Korea University College of Medicine, Ansan si 15355, Korea; jaykim830@gmail.com (J.Y.K.); gksmfskdls@gmail.com (E.-t.J.); 2 Division of Infectious Diseases, Department of Internal Medicine, Ansan Hospital, Korea University College of Medicine, Ansan si 15355, Korea; cmcws@hanmail.net; 3 Division of Endocrinology and Metabolism, Department of Internal Medicine, Ansan Hospital, Korea University College of Medicine, Ansan si 15355, Korea; nhkendo@gmail.com; 4 Department of Radiology, Ansan Hospital, Korea University College of Medicine, Ansan si 15355, Korea; kiylee@korea.ac.kr

**Keywords:** COVID-19, chest X-ray, deep learning, convolutional neural network, Grad-CAM

## Abstract

According to recent studies, patients with COVID-19 have different feature characteristics on chest X-ray (CXR) than those with other lung diseases. This study aimed at evaluating the layer depths and degree of fine-tuning on transfer learning with a deep convolutional neural network (CNN)-based COVID-19 screening in CXR to identify efficient transfer learning strategies. The CXR images used in this study were collected from publicly available repositories, and the collected images were classified into three classes: COVID-19, pneumonia, and normal. To evaluate the effect of layer depths of the same CNN architecture, CNNs called VGG-16 and VGG-19 were used as backbone networks. Then, each backbone network was trained with different degrees of fine-tuning and comparatively evaluated. The experimental results showed the highest AUC value to be 0.950 concerning COVID-19 classification in the experimental group of a fine-tuned with only 2/5 blocks of the VGG16 backbone network. In conclusion, in the classification of medical images with a limited number of data, a deeper layer depth may not guarantee better results. In addition, even if the same pre-trained CNN architecture is used, an appropriate degree of fine-tuning can help to build an efficient deep learning model.

## 1. Introduction

CORONAVIRUS disease (COVID-19) has quickly become a global pandemic since it was first reported in December 2019, reaching approximately 21.3 million confirmed cases and 761,799 deaths as of 16 August 2020 [1]. Due to the highly infectious nature and unavailability of appropriate treatments and vaccines for the virus, early screening of COVID-19 is crucial to prevent the spread of the disease by the timely isolation of susceptive individuals and the proper allocation of limited medical resources.

Currently, reverse transcription polymerase chain reaction (RT-PCR) was introduced as the gold standard screening method for COVID-19 [2]. However, since the overall positive rate of RT-PCR, using nasal and throat swabs, is reported to be 60–70% [3], there is a risk that a false-negative patient may again act as another source of infection in a healthy community. Conversely, there have been reports of high sensitivity to COVID-19 screening in radiological tests such as chest computed tomography or chest X-ray (CXR) [3,4,5]. According to the reports on CXR characteristics of patients confirmed as the COVID-19 case, it demonstrated multi-lobar involvement and peripheral airspace opacities, which was most frequently demonstrated as ground-glass [6]. However, in the early stages of COVID-19, this ground-glass pattern may appear at the edges of the lung vessels, or as asymmetric diffused airspace opacities [7], it can be difficult to visually detect the characteristic patterns of COVID-19 from X-rays. Therefore, considering the fact that the number of suspected patients increases exponentially in contrast to the limited number of highly trained radiologists, the diagnostic supporting procedures, using an automated screening algorithm with a producing objective, reproducible, and scalable results, can speed up earlier precise diagnosis.

In recent years, deep learning (DL) technology, a specific field of artificial intelligence (AI) technology, has made remarkable advances in medical image analysis and diagnosis, and is considered to be a potentially powerful tool to solve such problems [8,9]. Despite the lack of available published data to date, DL approaches for the diagnosis of COVID-19 from CXR have been actively studied [10,11,12,13,14,15,16,17]. Because the available data are limited, previous research has focused on creating a new DL architecture based on deep convolutional neural networks (CNNs) for providing effective diagnosis algorithms. However, previous studies have focused only on the efficacy of the newly created network through comparison between different CNNs, so the effect of the layer depth, called scalability, and degree of fine-tuning of transfer learning with CNN has not been comparatively studied. Therefore, the main objective of this study was to further investigate the effect of layer depth on the same CNN architecture, and the degree of fine-tuning of transfer learning with the same CNN at the same hyper-parameters. Furthermore, by employing the gradient-weighted class activation map (Grad-CAM) [18,19], this study provided a visual interpretation explaining the feature characteristic region that the DL model has the most influence on classification prediction.

## 2. Materials and Methods

### 2.1. Experimental Design

**Figure 1:**
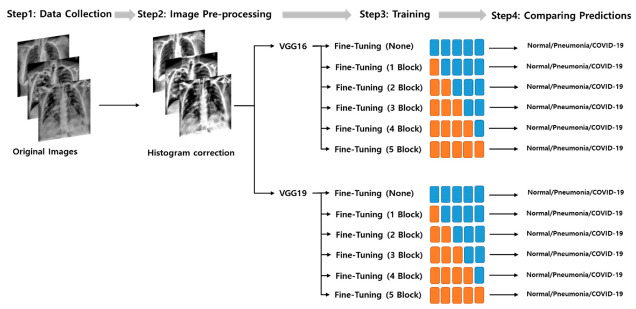
The experiment consists of a total of 12 experimental subgroups. It is largely divided into two main groups according to the layer depths, and each convolutional neural network (CNN) subgroup is divided into 6 subgroups according to the degree of fine-tuning.

The overall experimental steps and experimental groups used in this study are shown in Figure 1. The experiment consisted of 12 experimental subgroups. To evaluate the scalability of the same CNN architecture, the experiment consisted of two main groups according to the layer depths of each CNN. Each CNN main group is divided into 6 subgroups according to the degree of fine-tuning.

### 2.2. Datasets

The datasets used for classification are described in Table 1. Several publicly available image data repositories have been used to collect COVID-19 chest-ray images. Normal and pneumonia samples were extracted from the open source NIH chest X-ray dataset used for the Radiological Society of North America (RSNA) pneumonia detection challenge [20]. The total dataset was curated into three classes: normal, pneumonia, and COVID-19. Since the balance of data for each class is a very important factor in classification analysis, this study randomly extracted the images of other classes according to the number of COVID-19 images that can be obtained as much as possible.

The entire dataset was combined with 607 COVID-19 image data publicly shared at the time of the study, as well as 607 normal and 607 pneumonia chest radiographs randomly extracted from the RSNA Pneumonia Detection Challenge dataset, resulting in 1821 data being combined. In the case of the COVID-19 dataset, four public datasets were used, and only one image was used when the source of the image was duplicated. In the public datasets used in the experiment, patient information was de-identified or not provided.

The entire collected dataset was randomly divided into a training and testing ratio of 80:20 for each class, and training data were also randomly divided by a training and validation ratio of 80:20 for use in the 5-fold cross validation.

### 2.3. Image Preprocessing

**Figure 2:**
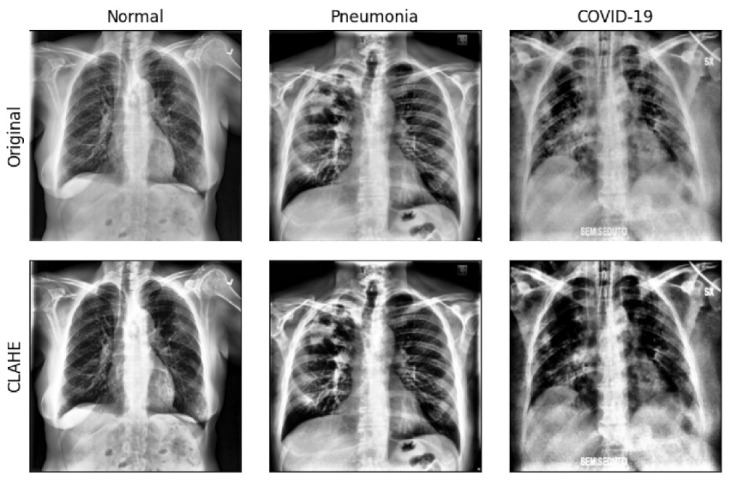
Sample images after applying contrast correction by contrast limited adaptive histogram equalization (CLAHE) and the semantic segmentation of lung on original chest X-ray (CXR) images.

Because the image data used in this experiment were collected from multiple centers, most of the images have different contrast and dimensions. Therefore, all images used in this study required contrast correction through the histogram equalization technique and resizing to a uniform size before the experiment. In this study, preprocessing was performed using the contrast limited adaptive histogram equalization (CLAHE) technique [25], which has been adopted in previous studies related to lung segmentation and pneumonia classification [26,27,28]. Figure 2 shows sample images with CXR contrast corrected using the CLAHE technique. For the consistency of image analysis, each image was resized to a uniform size of 800 × 800.

### 2.4. Convolutional Neural Networks

This study employed two different deep CNNs as backbone networks: VGG-16 and VGG-19. VGG [29] is a pre-trained CNN, from the Visual Geometry Group, Department of Engineering Science, University of Oxford. The numbers 16 and 19 represent the number of layers with trainable weights of VGG networks. VGG architecture had been widely adopted and recognized as a state of the art in both general and medical image classification tasks [30]. Since VGG-16 and VGG-19 have the same neural network architecture but different layer depths, a comparative evaluation of performance according to the degree of layer depths can be performed under the same architectural condition.

### 2.5. Fine-Tuning

**Figure 3:**
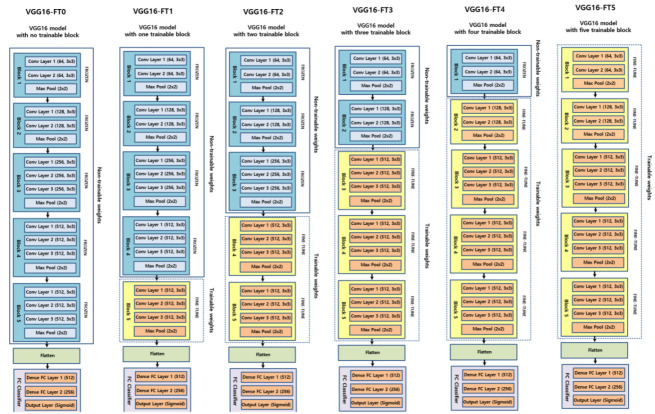

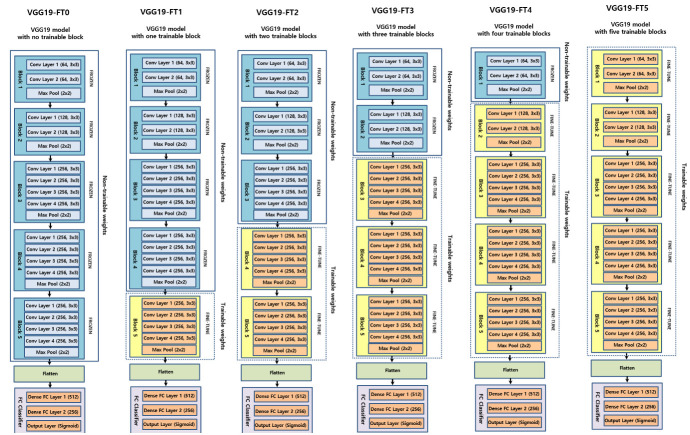
Schematic diagram of 12 experimental groups according to the degree of fine-tuning in the VGG-16 (**top**) and VGG-19 (**bottom**) backbone neural networks.

When the training dataset is relatively small, transferring a network pre-trained on a large annotated dataset and fine-tuning it for a specific task can be an efficient way to achieve acceptable accuracy and less training time [31]. Although the classification of diseases from CXR images differs from object classification and natural images, they can share similar learned features [32]. During the fine-tuning of transfer learning with deep CNNs, model weights were initialized based on pre-training on a general image dataset, except that some of the last blocks were unfrozen so that their weights were updated in each training step. In this study, the VGG-16 and VGG-19, used in this study as a backbone neural network, consist of 5 blocks regardless of the network layer depth. Therefore, fine-tuning was performed in a total of 6 steps in a manner that was unfrozen sequentially from 0 to 5 blocks starting from the last block, depending on how many blocks were unfrozen. As a result, VGG-16 and VGG-19 were used as backbone networks, and each deep CNN was divided into 6 subgroups according to the degree of fine-tuning. Figure 3 shows the schematic diagrams of the layer composition and the degree of fine-tuning of VGG-16 and VGG-19.

### 2.6. Training

**Figure 4:**
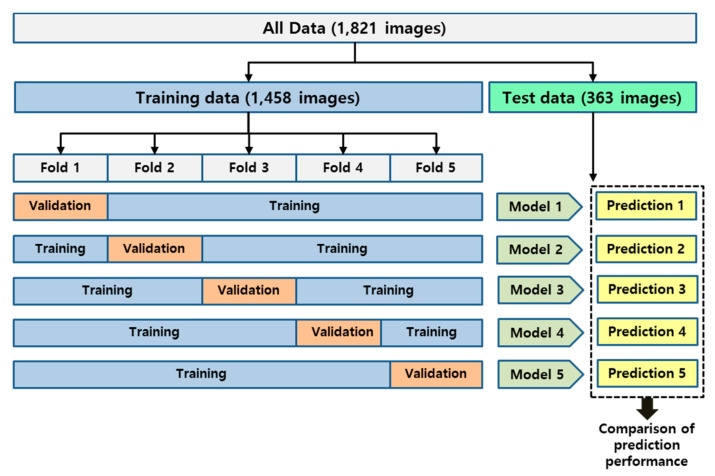
The overview of the 5-fold cross validation applied in this study.

The 1458 images selected as the training dataset were randomly divided into five folds. This was done to perform 5-fold cross validation to evaluate the model training, while avoiding overfitting or bias [33,34,35]. Within each fold, the dataset was partitioned into independent training and validation sets using an 80 to 20% split. The selected validation set was a completely independent fold from the other training folds and was used to evaluate the training status during the training. After one model training step was completed, the other independent fold was used as a validation set and the previous validation set was reused as part of the training set to evaluate the model training. An overview of the 5-fold cross validation performed in this study is presented in Figure 4. As an additional method to prevent overfitting, drop out was applied to the last fully connected layers, and early stopping was also applied by monitoring the validation loss at each epoch.

The above training process was repeated for all 24 experimental groups (Figure 1). All deep CNN models were trained and evaluated on an NVIDIA DGX StationTM (NVIDIA Corp., Santa Clara, CA, USA) with an Ubuntu 18 operating system, 256 GB system memory, and four NVIDIA Telsa V100 GPU. The building, training, validation, and prediction of DL models were performed using the Keras [36] library and TensorFlow [37] backend engine. The initial training rate of each model was 0.00001. A ReduceLROn-Plateau method was employed because it reduces the learning rate when it stops improving the training performance. The RMSprop algorithm was used as the solver. After training all the 5-fold deep CNN models, the best model was identified by testing with the test dataset.

### 2.7. Performance Evaluation

To comprehensively evaluate the screening performance on the test dataset, the accuracy, sensitivity, specificity, receiver operating characteristic (ROC) curve, and precision recall (PR) curve were calculated. The accuracy, sensitivity, and specificity score can be calculated as follows:

$$\mathrm{Accuracy}=\;\frac{TP+TN}{TP+TN+FN+FP}$$

$$\mathrm{Sensitivity}=\;\frac{TP}{TP+FN}$$

$$\mathrm{Specificity}=\;\frac{TN}{TN+FP}.$$

*TP* and *FP* are the number of correctly and incorrectly predicted images, respectively. Similarly, *TN* and *FN* represent the number of correctly and incorrectly predicted images, respectively. The area under the ROC curve (AUC) was also calculated in this study.

### 2.8. Interpretation of Model Prediction

Because it is difficult to know the process of how deep CNNs make predictions, DL models have often been referred to as non-interpretable black boxes. To determine the decision-making process of the model, and which features are most important for the model to screen COVID-19 in CXR images, this study employed the gradient-weighted class activation mapping technique (Grad-CAM) [18,19] so that the most significant regions for screening COVID-19 in CXR images were highlighted.

## 3. Results

### 3.1. Classification Performance

Table 2 summarizes the classification performance of the three classes, normal (N), pneumonia (P), and COVID-19 (C), for each experimental group.

Compared with all the tested deep CNN models, the fine-tuned with two blocks of the VGG-16 (VGG16-FT2) model achieved the highest performance in terms of the COVID-19 classification of accuracy (95.9%), specificity (97.5%), sensitivity (92.5%), and AUC (0.950). For all the tested deep CNNs, fine-tuning the last two convolutional blocks presented a higher classification performance compared to the fine-tuning of the other number of convolutional blocks. In addition, the case of all untrainable convolutional blocks without fine-tuning, regardless of the scalability of the backbone network, showed the lowest classification. Generally, the fine-tuned models using VGG16 as a backbone architecture were better than those using VGG19.

**Figure 5:**
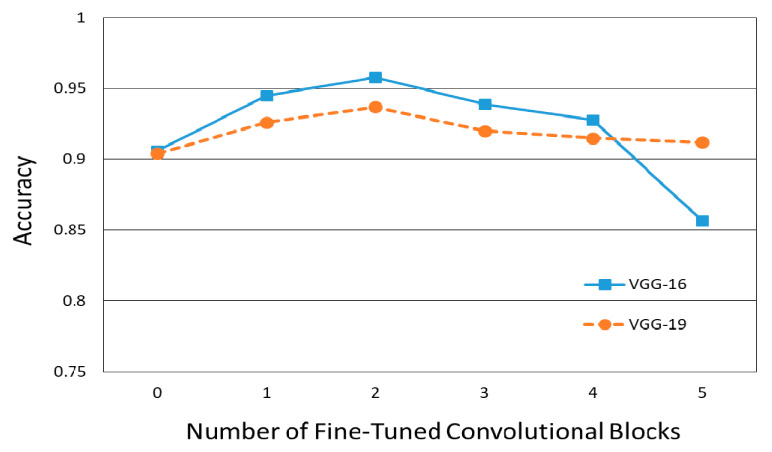
COVID-19 classification performance versus the number of fine-tuned convolutional blocks.

**Figure 6:**
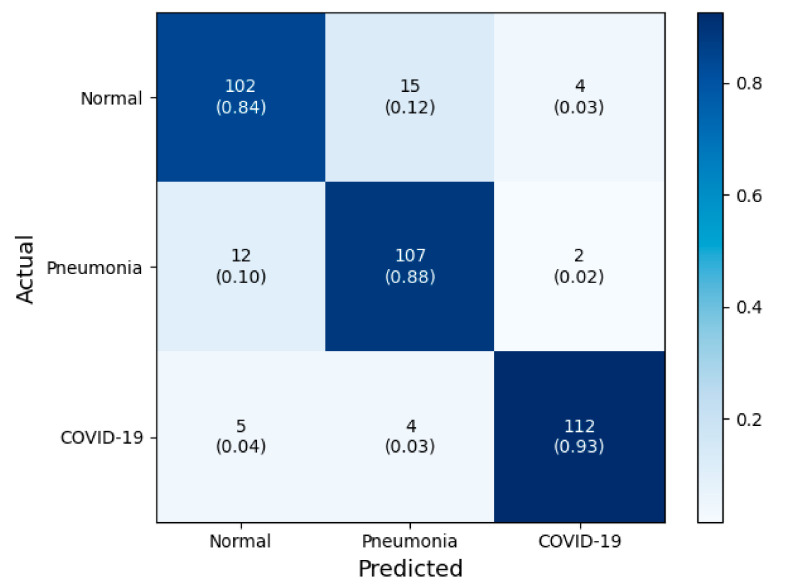
Confusion matrix of the best performed classification model (VGG16-FT2) in this study.

**Figure 7:**
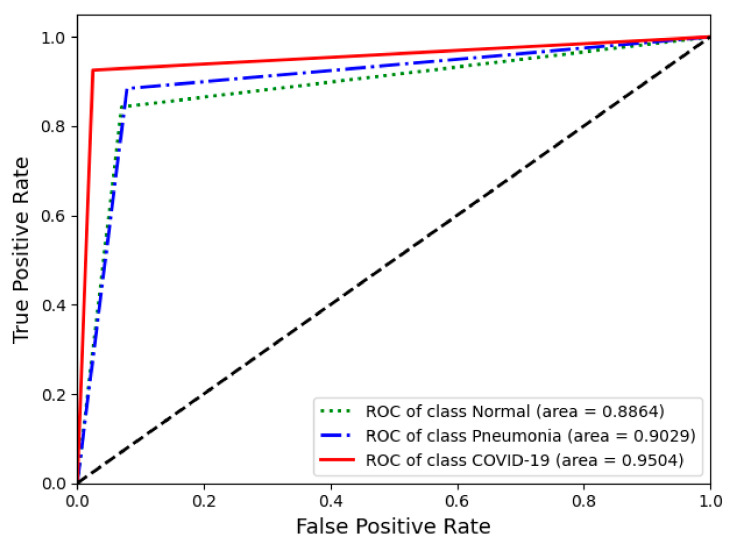
Receiver operating characteristics (ROC) curve of the best performing classification model (VGG16-FT2) in this study.

Figure 5 shows how the number of fine-tuned deep CNN blocks influences the classification performance in terms of the accuracy of COVID-19 screening. In this figure, the classification performance was not proportionately dependent on the degree of fine-tuning with the base model. There was a decrease in classification accuracy when more than three convolutional blocks of all deep CNNs were used. In addition, regardless of the number of fine-tuned blocks, the VGG19 models with more convolutional layers had lower classification accuracy than the VGG16 models. The confusion matrix and ROC of VGG16-FT2 achieving the highest performance in multi-class classification are presented in Figure 6 and Figure 7.

### 3.2. Interpretation of Model Decision Using Grad-CAM

**Figure 8:**
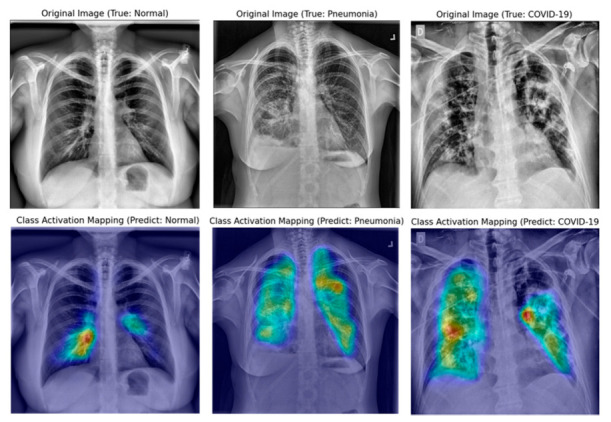
Samples of original and gradient-weighted class activation mapping technique (Grad-CAM) images were correctly predicted by the best performing classification model (VGG16-FT2) in this study.

**Figure 9:**
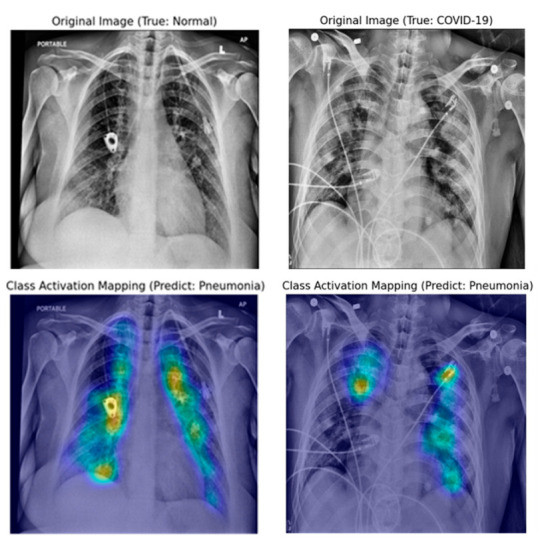
Original and Grad-CAM sample images presumed to be misclassified according to the wrong reason by the best performing classification model in this study (VGG16-FT2).

**Figure 10:**
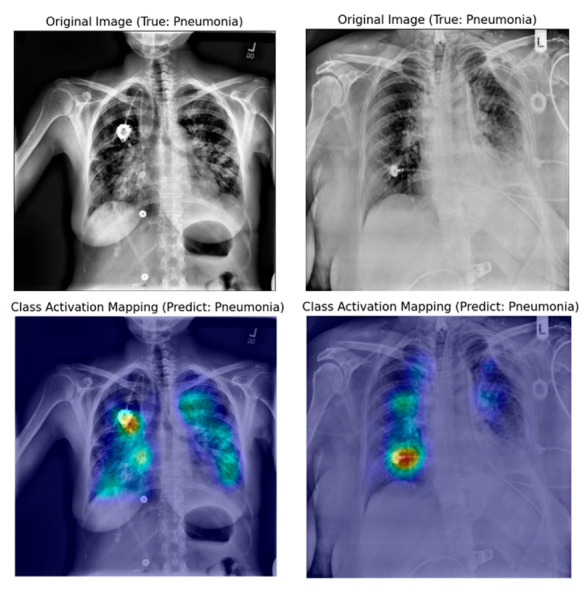
Original and Grad-CAM sample images presumed to be correctly classified according to the wrong reason by the best performing classification model in this study (VGG16-FT2).

Figure 8, Figure 9 and Figure 10 show examples of a visualized interpretation of predictions using deep CNN models in this study. In each example, the color heat map presented which areas were most affected by the classification of the deep CNN model.

Figure 8 shows representative examples of correctly classified cases for each of the three classes (normal, pneumonia, and COVID-19) in the VGG16-TF2 experimental group that showed the highest classification performance. Through the Grad-CAM result in Figure 8, it is possible to identify the significant region where the difference in CXR image features of each of the three classes is made. Figure 9 and Figure 10 show representative examples of wrong and right classifications based on the wrong reasons. In most cases where classification has occurred based on the wrong reason, there is a foreign body in the chest cavity of the CXR image.

## 4. Discussion

In addition to the long-term sustainability of the COVID-19 pandemic and symptom similarity with other pneumonia diseases, the limited medical resources and lack of expert radiologists have greatly increased the importance of screening for COVID-19 from CXR images for the right concentration of medical resources and isolation of potential patients. To overcome these limitations, various cutting-edge artificial intelligence (AI) technologies have been applied to screen COVID-19 from various medical data. Accordingly, until recently, numerous new DL models, such as COVID-Net [10], Deep-COVID [16], CVDNet [38], and Covid-resnet [13], to classify COVID-19 through publicly shared CXR images have been proposed, or mutual comparison studies through the transfer learning of various pre-trained DL models have been presented [39,40]. These previous papers showed high accuracy of more than 95%. However, most of them performed transfer learning but did not mention the specific degree of fine-tuning. It is also rare to have a qualitative evaluation. As a result, it is often difficult to reproduce a similar degree of accuracy with the same pre-trained DL model. Therefore, in the present study, the effects of the degree of fine-tuning and layer depths on deep CNNs for the screening performance of COVID-19 from CXR images were evaluated. Furthermore, these influences were visually interpreted using the Grad-CAM technique.

### 4.1. Scalability of Deep CNN

It is known that the VGG architecture used as the deep CNN backbone network in this experiment does not leverage residual principles, has a lightweight design, and low architectural diversity, so it is convenient to fine-tune [10]. In particular, the VGG-16 and VGG-19 used in this study have the same architecture with five convolutional blocks; however, the depth of the layers of VGG-19 is deeper than that of VGG-16 (Figure 3). 

According to Table 2 and Figure 5, the overall classification performance of VGG-16 was higher than that of VGG-19, regardless of the fine-tuning degree. These results are similar to the fact that the latest deep neural networks do not guarantee higher accuracy in the classification of medical images such as CXR images, as in other previous research papers [39]. It can be considered that in the case of medical images requiring less than 10 classifications, deep CNNs with low scalability can show better performance, unlike the classification of general objects that require more than 1000 classifications.

### 4.2. Degree of Fine-Tuning of Deep CNN

In general, the deep CNN model learned from pre-trained deep neural networks on a large natural image dataset which could be used to classify common images but cannot be well utilized for specific classifying tasks of medical images. However, according to a previous study that described the effects and mechanisms of fine-tuning on deep CNNs, when certain convolutional blocks of a deep CNN model were fine-tuned, the deep CNN model could be further specialized for specific classifying tasks [32,41]. More specifically, the earlier layers of a deep CNN contain generic features that should be useful for many classification tasks; however, later layers progressively contain more specialized features to the details of the classes contained in the original dataset. Using this property, when the parameters of the early layers are preserved and that in later layers are updated during the training of new datasets, the deep CNN model can be effectively used in new classification tasks. In conclusion, fine-tuning uses the parameters learned from a previous training of the network on a large dataset, and then adjusts the parameters in later layers from the new dataset, improving the performance and accuracy in the new classification task.

As far as the authors know, there has been no previous research paper evaluating the accuracy of COVID-19 screening according to the degree of fine-tuning. According to Figure 5, regardless of the scalability of VGG, classification accuracy increases as the degree of fine-tuning increases; however, the fine-tuning of more than a certain convolutional block (more than 3 blocks in this experiment) decrease the classification accuracy. Therefore, it seems necessary to find the appropriate degree of fine-tuning by judging the degree of fine-tuning in the transfer learning by a hyper-parametric variable such as batch-size or learning rate in DL.

### 4.3. Visual Interpretation Using Grad-CAM

Grad-CAM uses the gradient information flowing into the last convolutional layer of the deep CNN to understand the significance of each neuron for making decisions [18]. In this experiment, a qualitative evaluation of classification adequacy was performed using the Grad-CAM technique. In the case of the deep CNN model, which showed the best classification as shown in Figure 8, image feature points for each class were specified within the lung cavity in CXR images. However, as shown in Figure 9, if there is a foreign substance in the lung cavity in a CXR image, it can be classified incorrectly. Moreover, even if a CXR image is correctly classified, it can be classified for an incorrect reason as shown in Figure 10. In the CXR image analysis using the DL algorithm, the implanted port catheter and pacemaker or defibrillator generator have shown similar results to the previous studies that interfere with the performance of the DL algorithm by causing false positives or false negatives [42]. This shows the pure function of the Grad-CAM technique and suggests candidate areas to be excluded through image preprocessing for areas or foreign body subjects that affect classification accuracy improvement on the image.

## 5. Conclusions

This experiment showed the appropriate transfer learning strategy of a deep CNN to screen for COVID-19 in CXR images as follows. In using the deep CNNs for COVID-19 screening in CXR images, it is not always guaranteed to achieve cutting-edge results, increasing their complexity and layer depth. In addition, when applying transfer learning to a deep CNN for classification, an appropriate degree of fine-tuning is required, and this must also be treated as an important hyper-parametric variable that affects the accuracy of DL. In particular, in the case of image classification using DL, it is also necessary to qualitatively evaluate a classification as to whether an appropriate classification has occurred based on the correct reason, using visual interpretation methods such as the Grad-CAM technique.

## Figures and Tables

**Table 1:** Description of datasets for COVID-19 classification.

Class	Reference	Samples
Normal	RSNA pneumonia detection challenge [20]	607
Pneumonia	RSNA pneumonia detection challenge [20]	607
COVID-19	COVID-19 image data collection [21]	468
	Figure 1 COVID-19 Chest X-ray [22]	35
	Actualmed COVID-19 Chest X-rays [23]	58
	COVID-19 Radiography Database [24]	46
Total		1821

**Table 2:** Performance metrics of experimental groups where N, P and C are normal, pneumonia and COVID-19, respectively.

CNN Models	Number of Fine-Tuning Blocks		Accuracy	Specificity	Sensitivity	AUC
VGG-16	0	N	0.871	0.909	0.793	0.851
		P	0.832	0.814	0.868	0.841
		C	0.906	0.883	0.752	0.868
	1	N	0.873	0.884	0.851	0.868
		P	0.884	0.913	0.826	0.870
		C	0.945	0.979	0.876	0.928
	2	N	0.901	0.930	0.842	0.886
		P	0.909	0.921	0.884	0.903
		C	0.959	0.975	0.925	0.950
	3	N	0.884	0.888	0.876	0.882
		P	0.884	0.909	0.835	0.872
		C	0.939	0.983	0.851	0.917
	4	N	0.901	0.934	0.835	0.884
		P	0.862	0.847	0.893	0.870
		C	0.928	0.988	0.810	0.899
	5	N	0.873	0.905	0.810	0.857
		P	0.796	0.748	0.893	0.820
		C	0.857	0.992	0.587	0.789
VGG-19	0	N	0.873	0.971	0.678	0.824
		P	0.804	0.777	0.860	0.818
		C	0.904	0.938	0.835	0.886
	1	N	0.893	0.913	0.851	0.882
		P	0.857	0.893	0.785	0.836
		C	0.926	0.950	0.876	0.913
	2	N	0.882	0.909	0.826	0.868
		P	0.868	0.905	0.793	0.849
		C	0.937	0.950	0.909	0.930
	3	N	0.879	0.897	0.843	0.870
		P	0.847	0.876	0.777	0.826
		C	0.920	0.959	0.843	0.901
	4	N	0.860	0.872	0.835	0.853
		P	0.840	0.876	0.769	0.822
		C	0.915	0.963	0.818	0.890
	5	N	0.862	0.864	0.860	0.862
		P	0.835	0.888	0.727	0.808
		C	0.912	0.955	0.826	0.890

## References

[1] World Health Organization (2020). Coronavirus Disease (COVID-19): Situation Report, 182.

[2] Wang W., Xu Y., Gao R., Lu R., Han K., Wu G., Tan W. (2020). Detection of SARS-CoV-2 in different types of clinical specimens. JAMA.

[3] Yang Y., Yang M., Shen C., Wang F., Yuan J., Li J., Zhang M., Wang Z., Xing L., Wei J. (2020). Laboratory diagnosis and monitoring the viral shedding of 2019-nCoV infections. MedRxiv.

[4] Kanne J.P., Little B.P., Chung J.H., Elicker B.M., Ketai L.H. (2020). Essentials for Radiologists on COVID-19: An Update—Radiology Scientific Expert Panel.

[5] Ai T., Yang Z., Hou H., Zhan C., Chen C., Lv W., Tao Q., Sun Z., Xia L. (2020). Correlation of chest CT and RT-PCR testing in coronavirus disease 2019 (COVID-19) in China: A report of 1014 cases. Radiology.

[6] Kong W., Agarwal P.P. (2020). Chest imaging appearance of COVID-19 infection. Radiol. Cardiothorac. Imaging.

[7] Rodrigues J., Hare S., Edey A., Devaraj A., Jacob J., Johnstone A., McStay R., Nair A., Robinson G. (2020). An update on COVID-19 for the radiologist—A British society of Thoracic Imaging statement. Clin. Radiol..

[8] Zhou S.K., Greenspan H., Shen D. (2017). Deep Learning for Medical Image Analysis.

[9] Shen D., Wu G., Suk H.-I. (2017). Deep learning in medical image analysis. Annu. Rev. Biomed. Eng..

[10] Wang L., Wong A. (2020). COVID-Net: A Tailored Deep Convolutional Neural Network Design for Detection of COVID-19 Cases from Chest X-ray Images. arXiv.

[11] Narin A., Kaya C., Pamuk Z. (2020). Automatic detection of coronavirus disease (covid-19) using X-ray images and deep convolutional neural networks. arXiv.

[12] Hemdan E.E.-D., Shouman M.A., Karar M.E. (2020). Covidx-net: A framework of deep learning classifiers to diagnose covid-19 in X-ray images. arXiv.

[13] Farooq M., Hafeez A. (2020). Covid-resnet: A deep learning framework for screening of covid19 from radiographs. arXiv.

[14] Afshar P., Heidarian S., Naderkhani F., Oikonomou A., Plataniotis K.N., Mohammadi A. (2020). Covid-caps: A capsule network-based framework for identification of covid-19 cases from X-ray images. arXiv.

[15] Oh Y., Park S., Ye J.C. (2020). Deep learning covid-19 features on cxr using limited training data sets. IEEE Trans. Med Imaging.

[16] Minaee S., Kafieh R., Sonka M., Yazdani S., Jamalipour Soufi G. (2020). Deep-COVID: Predicting COVID-19 from chest X-ray images using deep transfer learning. Med. Image Anal..

[17] Apostolopoulos I.D., Mpesiana T.A. (2020). Covid-19: Automatic detection from X-ray images utilizing transfer learning with convolutional neural networks. Phys. Eng. Sci. Med..

[18] Selvaraju R.R., Cogswell M., Das A., Vedantam R., Parikh D., Batra D. Grad-cam: Visual explanations from deep networks via gradient-based localization. Proceedings of the IEEE International Conference on Computer Vision.

[19] Chattopadhay A., Sarkar A., Howlader P., Balasubramanian V.N. Grad-cam++: Generalized gradient-based visual explanations for deep convolutional networks. Proceedings of the 2018 IEEE Winter Conference on Applications of Computer Vision (WACV).

[20] Radiological Society of North America (2018). RSNA Pneumonia Detection Challenge.

[21] Cohen J.P., Morrison P., Dao L. (2020). COVID-19 image data collection. arXiv.

[22] Chung A. (2020). Figure 1 COVID-19 Chest X-ray Data Initiative. https://github.com/agchung/Figure1-COVID-chestxray-dataset.

[23] Chung A. (2020). Actualmed COVID-19 Chest X-ray Data Initiative. https://github.com/agchung/Actualmed-COVID-chestxray-dataset.

[24] Rahman T., Chowdhury M., Khandakar A. (2020). COVID-19 Radiography Database.

[25] Stark J.A. (2000). Adaptive image contrast enhancement using generalizations of histogram equalization. IEEE Trans. Image Process..

[26] Ferreira J.R., Cardenas D.A.C., Moreno R.A., de Sá Rebelo M.d.F., Krieger J.E., Gutierrez M.A. Multi-View Ensemble Convolutional Neural Network to Improve Classification of Pneumonia in Low Contrast Chest X-ray Images. Proceedings of the 2020 42nd Annual International Conference of the IEEE Engineering in Medicine & Biology Society (EMBC).

[27] Ntirogiannis K., Gatos B., Pratikakis I. (2014). A combined approach for the binarization of handwritten document images. Pattern Recognit. Lett..

[28] Singh R.K., Pandey R., Babu R.N. (2020). COVIDScreen: Explainable deep learning framework for differential diagnosis of COVID-19 using chest X-rays. Res. Sq..

[29] Simonyan K., Zisserman A. (2014). Very deep convolutional networks for large-scale image recognition. arXiv.

[30] Litjens G., Kooi T., Bejnordi B.E., Setio A.A.A., Ciompi F., Ghafoorian M., Van Der Laak J.A., Van Ginneken B., Sánchez C.I. (2017). A survey on deep learning in medical image analysis. Med. Image Anal..

[31] Yosinski J., Clune J., Bengio Y., Lipson H. How transferable are features in deep neural networks?. Proceedings of the Advances in Neural Information Processing Systems (NIPS 2014).

[32] Pan S.J., Yang Q. (2009). A survey on transfer learning. IEEE Trans. Knowl. Data Eng..

[33] Stone M. (1974). Cross-validatory choice and assessment of statistical predictions. J. R. Stat. Soc. Ser. B Methodol..

[34] Cawley G.C., Talbot N.L. (2010). On over-fitting in model selection and subsequent selection bias in performance evaluation. J. Mach. Learn. Res..

[35] Steyerberg E.W., Harrell F.E. (2016). Prediction models need appropriate internal, internal–external, and external validation. J. Clin. Epidemiol..

[36] Chollet F. (2018). Keras: The python deep learning library. ascl.

[37] Abadi M., Agarwal A., Barham P., Brevdo E., Chen Z., Citro C., Corrado G.S., Davis A., Dean J., Devin M. (2016). Tensorflow: Large-scale machine learning on heterogeneous distributed systems. arXiv.

[38] Ouchicha C., Ammor O., Meknassi M. (2020). CVDNet: A novel deep learning architecture for detection of coronavirus (Covid-19) from chest X-ray images. ChaosSolitons Fractals.

[39] Bressem K.K., Adams L., Erxleben C., Hamm B., Niehues S., Vahldiek J. (2020). Comparing Different Deep Learning Architectures for Classification of Chest Radiographs. arXiv.

[40] Marques G., Agarwal D., de la Torre Díez I. (2020). Automated medical diagnosis of COVID-19 through EfficientNet convolutional neural network. Appl. Soft Comput..

[41] Tajbakhsh N., Shin J.Y., Gurudu S.R., Hurst R.T., Kendall C.B., Gotway M.B., Liang J. (2016). Convolutional neural networks for medical image analysis: Full training or fine tuning?. IEEE Trans. Med. Imaging.

[42] Singh R., Kalra M.K., Nitiwarangkul C., Patti J.A., Homayounieh F., Padole A., Rao P., Putha P., Muse V.V., Sharma A. (2018). Deep learning in chest radiography: Detection of findings and presence of change. PLoS ONE.

